# Structural and Functional Enrichment Analyses for Antimicrobial Peptides

**DOI:** 10.3390/ijms21228783

**Published:** 2020-11-20

**Authors:** Sheng C. Lo, Zhong-Ru Xie, Kuan Y. Chang

**Affiliations:** 1 Computational Biology Laboratory, Department of Computer Science and Engineering, National Taiwan Ocean University, Keelung 202, Taiwan; sanjacklo0308@gmail.com; 2 School of Electrical and Computer Engineering, College of Engineering, University of Georgia, Athens, GA 30602, USA; paulxie@uga.edu

**Keywords:** structure–function relationships, enrichment analysis, antifungal activities, knottin, two-layer sandwich architecture

## Abstract

Whether there is any inclination between structures and functions of antimicrobial peptides (AMPs) is a mystery yet to be unraveled. AMPs have various structures associated with many different antimicrobial functions, including antibacterial, anticancer, antifungal, antiparasitic and antiviral activities. However, none has yet reported any antimicrobial functional tendency within a specific category of protein/peptide structures nor any structural tendency of a specific antimicrobial function with respect to AMPs. Here, we examine the relationships between structures categorized by three structural classification methods (CATH, SCOP, and TM) and seven antimicrobial functions with respect to AMPs using an enrichment analysis. The results show that antifungal activities of AMPs were tightly related to the two-layer sandwich structure of CATH, the knottin fold of SCOP, and the first structural cluster of TM. The associations with knottin and TM Cluster 1 even sustained through the AMPs with a low sequence identity. Moreover, another significant mutual enrichment was observed between the third cluster of TM and anti-Gram-positive-bacterial/anti-Gram-negative-bacterial activities. The findings of the structure–function inclination further our understanding of AMPs and could help us design or discover new therapeutic potential AMPs.

## 1. Introduction

Antimicrobial peptides (AMPs) or proteins have a broad spectrum of biological activities and various structures [1]. On one hand, these biological activities of AMPs include antibacterial, antifungal, antiparasitic, antiviral, and anticancer activities. On the other hand, various structures of natural AMPs, such as cathelicidin, defensin, and transferrin, have been resolved [2]. However, little is known about propensities between antimicrobial activities and AMP structures.

Attention has been paid to AMP structure–function relationships [3,4,5]. One well-studied example is defensins found in fungi, plants, and animals. Defensins have multiple intramolecular disulfide bonds between at least six conserved cysteine residues, which maintain their structures against chemical and proteolytic degradation and are crucial to their antimicrobial functions. Moreover, positively charged residues along with amphipathic characters within the structures have also been linked to their functions such as antifungal activities [6,7]. Although defensins exhibit a broad spectrum of antimicrobial activities, including antibacterial, antifungal, and antiviral activities, antifungal activities are mostly observed, especially for plant defensins [6,7]. However, none demonstrate any functional tendency toward any structural groups of AMPs, like defensins, nor any structural tendency toward any antimicrobial activities of AMPs.

To establish the associations between antimicrobial activities and AMP structures, we perform large-scale systematic enrichment analyses based on three kinds of structural classification (CATH (class, architecture, topology, homology) [8], SCOP (structural classification of proteins) [9], and TM (template modeling) [10]. This study broadens our understanding of AMP structure–function relationships, which could benefit finding or designing therapeutic potential AMPs as peptide antibiotics.

## 2. Results

### 2.1. Enriched AMP Functions

#### 2.1.1. Enriched AMP Functions in Terms of CATH Structures

**Figure 1:**
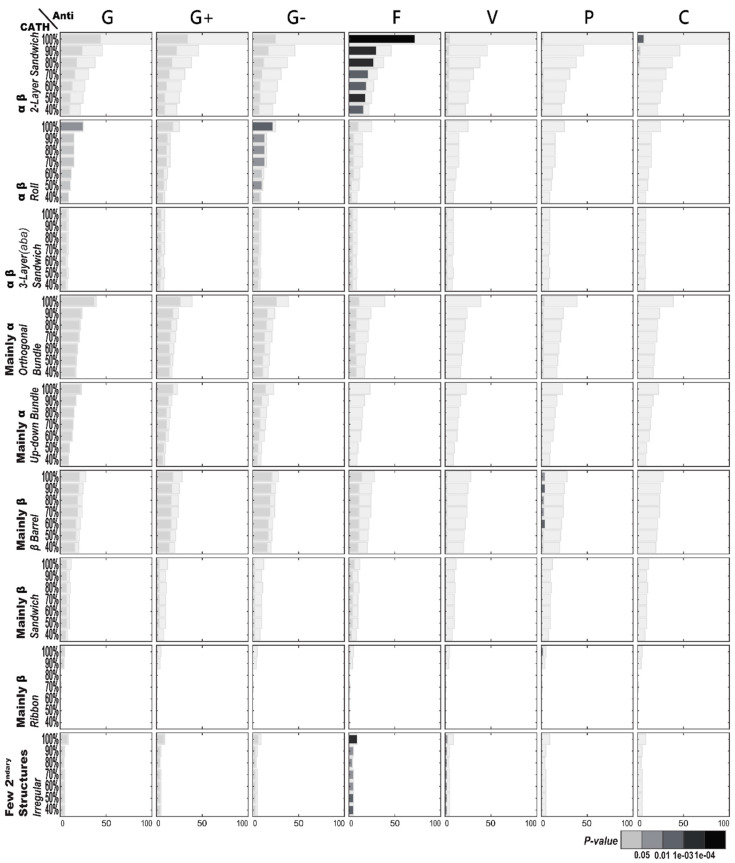
Antimicrobial peptide (AMP) functional enrichment analysis with respect to CATH structures. The *X*-axis and *Y*-axis are, respectively, the number of AMP sequences and the percentage of sequence identity. The bar in light gray indicates the potential maximum number of AMP sequences with the specific function. Abbreviations: C, anticancer; F, antifungal; G, antibacterial; G+, anti-Gram+; G-, anti-Gram-; P, antiparasitic; V, antiviral.

Figure 1 illustrates the functional enrichment analysis of AMPs with regard to CATH structures. All of these specific CATH structures, except the two-layer sandwich architecture under the α-β class, contain a majority of AMPs that exhibit antibacterial activities. Particularly, antibacterial activities are found to predominate in both the orthogonal bundle architecture of the mainly α class and the roll architecture of the α-β class of the AMPs, but neither pass the stringent enrichment tests. The roll architecture of the α-β class is also weakly associated with anti-Gram-negative bacterial activities. Despite widespread antibacterial activities, only antifungal activities within two-layer sandwich structures occur significantly more than random. In fact, the enriched antifungal activities are so significant within two-layer sandwich structures that the functional enrichment remains for the structures under 40% sequence identity.

#### 2.1.2. Enriched AMP Functions in Terms of SCOP Structures

**Figure 2:**
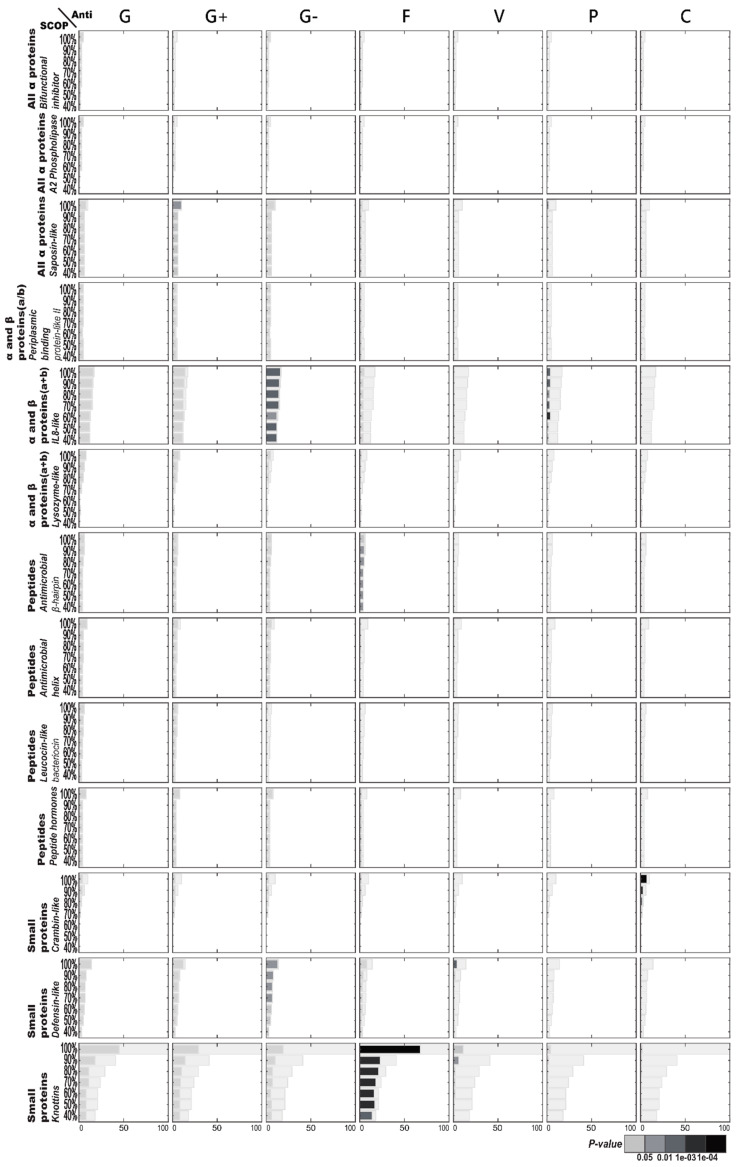
Antimicrobial peptide (AMP) functional enrichment analysis with respect to SCOP structures. The *X*-axis and *Y*-axis are, respectively, the number of AMP sequences and the percentage of sequence identity. The bar in light gray indicates the potential maximum number of AMP sequences with the specific function. Abbreviations: C, anticancer; F, antifungal; G, antibacterial; G+, anti-Gram+; G-, anti-Gram-; P, antiparasitic; V, antiviral.

Figure 2 illustrates the functional enrichment analysis of AMPs with regard to SCOP structures. There are more SCOP structures corresponding to AMPs than CATH structures, resulting in fewer members on average, with a specific function per category of the SCOP structures. Like the CATH structures, these specific SCOP structures generally exhibit antibacterial activities. Anti-Gram-negative bacterial activities are frequently observed for both IL8-like and defensin-like structures, but these enrichments are not strong enough to pass the correction for multiple hypotheses. There are also enriched functions found in a few other structures, but usually limited to the AMPs with over 90% sequence identity. For example, enriched antiviral activities are detected only in the highly similar AMPs of the defensin-like fold. Another observation worth mentioning is that enriched anticancer activities are associated with the AMPs within the crambin-like fold. However, only enriched antifungal activities sustain statistical significance through the knottins fold of small proteins with a low sequence similarity.

#### 2.1.3. Enriched AMP Functions in Terms of TM Structures

**Figure 3:**
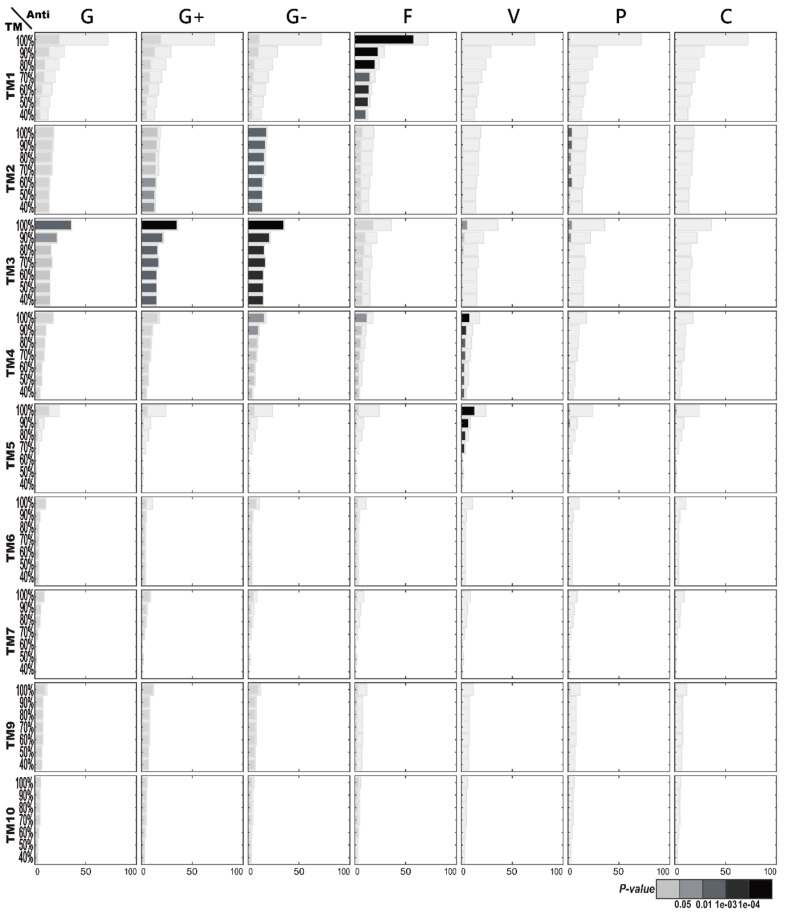
Antimicrobial peptide (AMP) functional enrichment analysis with respect to TM (template modeling) structures. The *X*-axis and *Y*-axis are, respectively, the number of AMP sequences and the percentage of sequence identity. The bar in light gray indicates the potential maximum number of AMP sequences with the specific function. TM8 was discarded because only the structural clusters which contain at least five AMPs are displayed here. Abbreviations: C, anticancer; F, antifungal; G, antibacterial; G+, anti-Gram+; G-, anti-Gram-; P, antiparasitic; V, antiviral.

Figure 3 illustrates the functional enrichment analysis of AMPs with regard to TM structures. Interestingly, more enriched functions are found on TM structural clusters than on CATH or SCOP structures of the AMPs. Most noticeably, enriched antifungal activities are significantly present in TM Cluster 1. Moreover, enriched anti-Gram-positive and anti-Gram-negative bacterial activities are demonstrated within TM Cluster 3. Other less significant functional enrichments include enriched antiviral activities in both TM Cluster 4 and Cluster 5; enriched anti-Gram-negative bacterial activities that are weakly connected to TM Cluster 2; anti-Gram-positive bacterial activities that are frequently observed in TM Cluster 2. 

### 2.2. Enriched AMP Structures

#### 2.2.1. Enriched CATH Structures in Terms of AMP Functions

**Figure 4:**
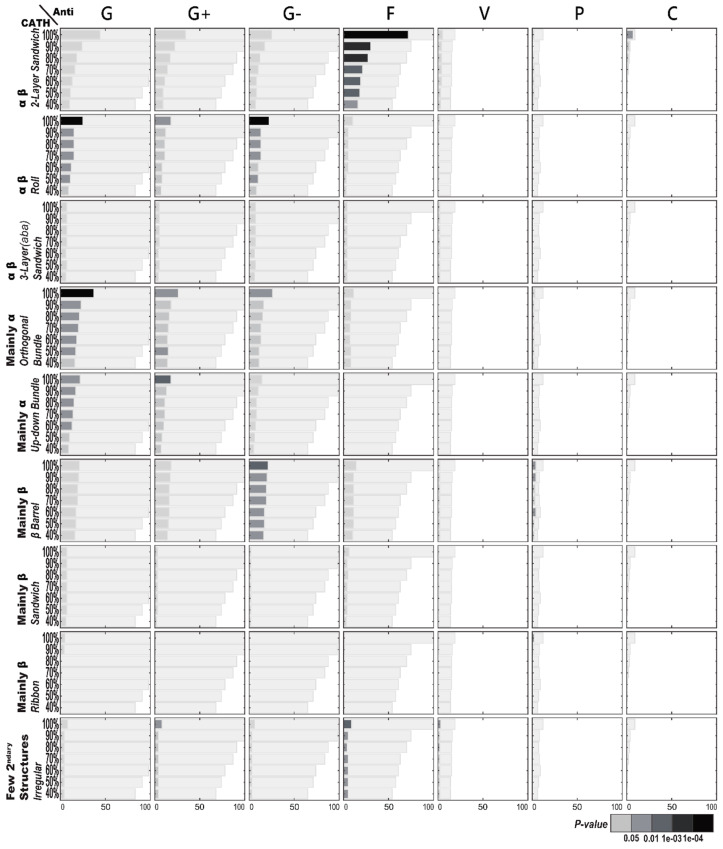
CATH structural enrichment analysis of antimicrobial peptides (AMPs) with respect to their functions. The *X*-axis and *Y*-axis are, respectively, the number of AMP sequences and the percentage of sequence identity. The bar in light gray indicates the potential maximum number of AMP sequences with the specific structure. Abbreviations: C, anticancer; F, antifungal; G, antibacterial; G+, anti-Gram+; G-, anti-Gram-; P, antiparasitic; V, antiviral.

Figure 4 illustrates the structural enrichment analysis using CATH structural classification with regard to specific AMP functions. Different from the functional enrichment above, the structural enrichment provides a distinct view of the AMPs. Among these examined AMP functions, only antifungal activities of the AMPs consistently express a structural inclination—the two-layer sandwich architecture under the α-β class is a clear favorite among all the CATH structures of the AMPs with antifungal activities. As to the AMPs with antibacterial activities, the orthogonal bundle and up–down bundle architectures of the mainly α class, as well as the roll architecture of the α-β class, occur more frequently than random. Yet these structural enrichments are still not as strong as the two-layer sandwich architecture to antifungal activities, whose structural enrichment remains through low sequence identity. As to the AMPs with anti-Gram-negative bacterial activities, the β barrel architecture of the mainly β class is frequently observed; so is the roll architecture of the α-β class. However, the enrichment of the roll architecture is slightly weaker than that of the β barrel architecture under low sequence identity.

#### 2.2.2. Enriched SCOP Structures in Terms of AMP Functions

**Figure 5:**
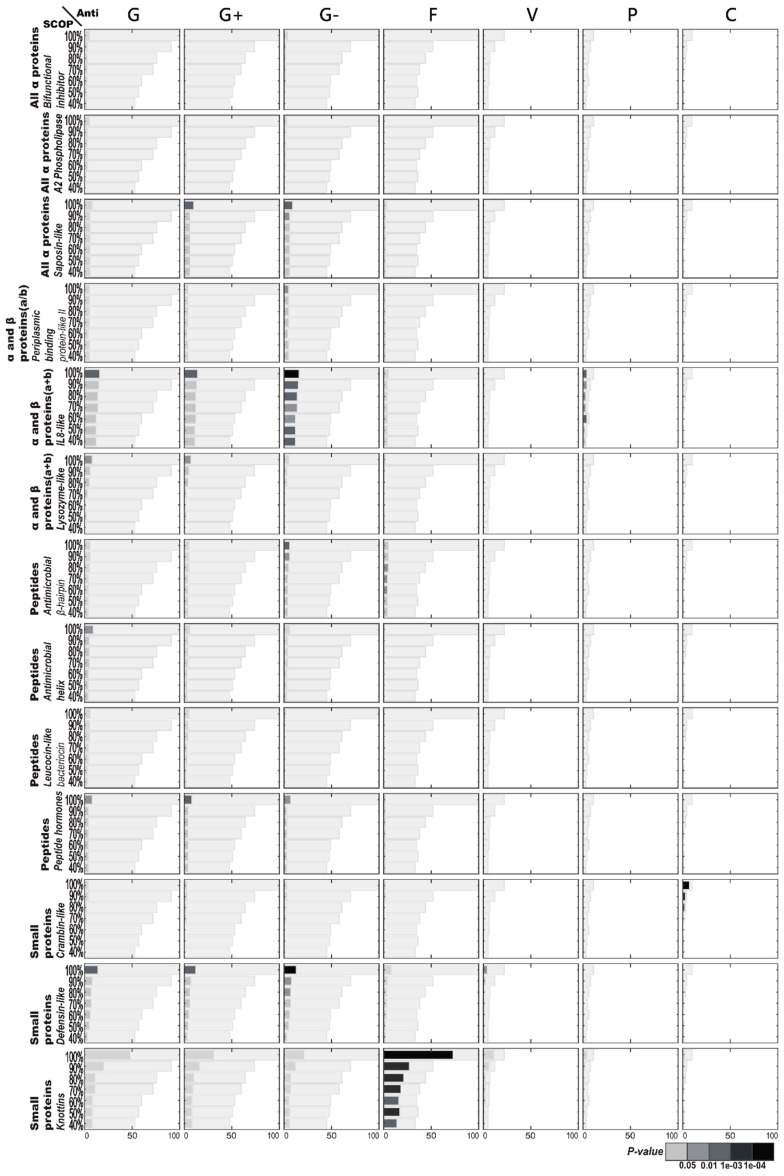
SCOP structural enrichment analysis of antimicrobial peptides (AMPs) with respect to their functions. The *X*-axis and *Y*-axis are, respectively, the number of AMP sequences and the percentage of sequence identity. The bar in light gray indicates the potential maximum number of AMP sequences with the specific structure. Abbreviations: C, anticancer; F, antifungal; G, antibacterial; G+, anti-Gram+; G-, anti-Gram-; P, antiparasitic; V, antiviral.

Figure 5 illustrates the structural enrichment analysis of SCOP structures with regard to specific AMP functions. Considering the AMPs with antifungal activities, the knottin fold of small proteins is found to be significantly enriched. Knottins are one of very few SCOP structures that can maintain the significance of structural enrichment under 40% sequence identity. Like knottins among the antifungal AMPs, the IL8-like fold of α and β proteins (α + β) is enriched among the anti-Gram-negative bacterial AMPs. Moreover, the defensin-like fold of small proteins occurs slightly more than random among antibacterial AMPs. Other less apparent structural enrichments include the saposin-like fold of all α proteins and the antimicrobial β hairpin of peptides among the AMPs with anti-Gram-negative activities, as well as the crambin-like fold of small proteins among those with anticancer activities.

#### 2.2.3. Enriched TM Structures in Terms of AMP Functions

**Figure 6:**
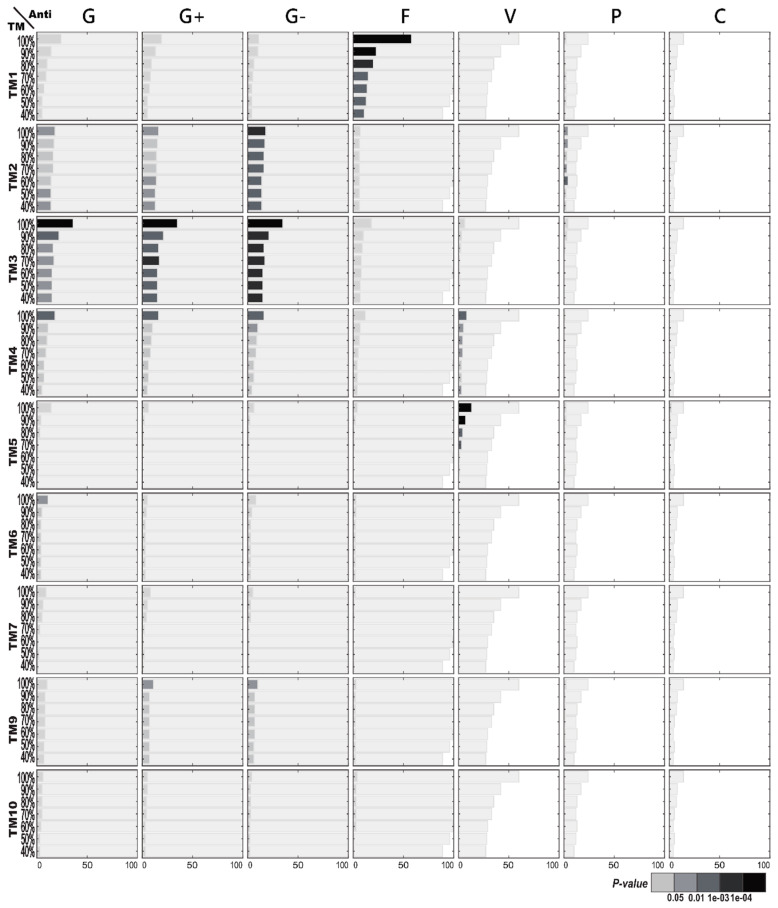
TM (template modeling) structural enrichment analysis of antimicrobial peptides (AMPs) with respect to AMP functions. The *X*-axis and *Y*-axis are, respectively, the number of AMP sequences and the percentage of sequence identity. The bar in light gray indicates the potential maximum number of AMP sequences within the specific TM cluster. TM8 was discarded because only the structural clusters that contain at least five AMPs are displayed here. Abbreviations: C, anticancer; F, antifungal; G, antibacterial; G+, anti-Gram+; G-, anti-Gram-; P, antiparasitic; V, antiviral.

Figure 6 illustrates the structural enrichment analysis following TM structural classification with regard to specific AMP functions. Most significantly, TM Structural Cluster 1 is enriched among the AMPs with antifungal activities. TM Clusters 3 and 2 are both enriched among the AMPs with antibacterial, anti-Gram-positive or anti-Gram-negative bacterial activities, especially with anti-Gram-negative bacterial activities, while the enrichment toward the TM3 cluster is stronger than toward the TM2 cluster. Similarly, both TM Clusters 4 and 5, which are less abundant than TM1, TM2, or TM3, are enriched among the AMPs with antiviral activities.

### 2.3. AMP Structure–Function Enrichment Analysis 

Table 1 shows the important enrichment relationships between antimicrobial functions and AMP structures categorized by the three kinds of structural classification (CATH, SCOP, and TM). Basically, Table 1 compiles the functional and structural enrichments of Figure 1, Figure 2, Figure 3, Figure 4, Figure 5 and Figure 6, but imposes more stringent requirements. Table 1 is restricted to those which contain at least 10 original AMP sequences per structural category and satisfy the additional Benjamini–Hochberg correction [11]. The most noticeable mutual enrichments happen between antifungal activities and the SCOP Small Proteins Class and between anti-Gram-negative bacterial activities and TM Structural Cluster 3. Even under low sequence similarity, with ≤40% sequence identity, the mutual relationships remain. 

Antifungal activities are well associated, with one structure categorized by either CATH, SCOP, or TM. Antifungal activities are persistently enriched in the SCOP Knottin Fold of Small Proteins and TM Structural Cluster 1 under ≤50% sequence identity. The mutual enrichment involved with antifungal activities also occurs in the CATH two-layer sandwich architecture under highly similar AMP sequences (≤80% sequence identity).

Some mutual enrichments, other than those involved with antifungal activities, exist. The structure–function mutual tendencies are observed between TM Structural Cluster 2 and anti-Gram-negative bacterial activities and between TM Cluster 3 and anti-Gram-positive bacterial activities. In addition, weak mutual enrichments include the following: anticancer activities and Crambin-like fold; antiviral activities and TM Cluster 5.

As to one-direction enrichment, TM Cluster 4 possesses enriched antiviral activities, but antiviral activities do not prefer to be in TM Cluster 4; antibacterial activities favor mainly alpha proteins of CATH, but alpha proteins need not possess antibacterial activities.

There are several functional enrichments or structural enrichments for the representative AMPs at 100% sequence identity but not at other thresholds. For example, anti-Gram-negative-bacterial activities are enriched in defensin-like structures for the original AMPs; mainly beta proteins of CATH have more antiparasitic activities than expected.

### 2.4. AMP Structure–Function Enrichments Associated with Sequences 

**Figure 7:**
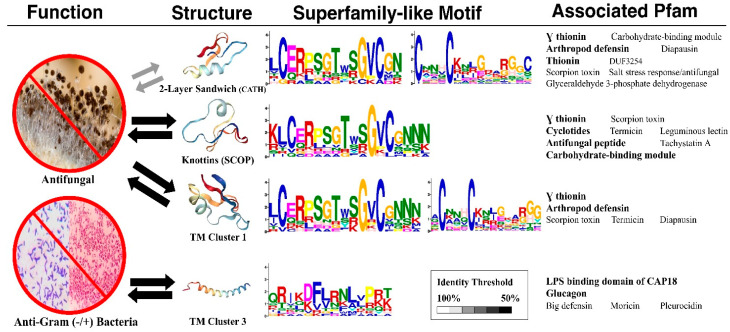
AMP structure–function enrichments associated with sequence motifs and Pfams.

Figure 7 illustrates the sequence motifs and associated Pfams for the AMP structure–function enrichments. According to the biological activities of AMPs, two kinds of structure–function enrichments remain mutually significant at 50% sequence identity: one associated with antifungal activities and the other with anti-Gram-negative/anti-Gram-positive activities.

#### 2.4.1. Superfamily-Like Sequence Motifs

The sequence motifs of the structure–function enrichments associated with antifungal activities are similar, albeit with some differences. All the motifs display multiple conserved cysteine residues. In primary motifs, a distinct glycine that is beside a conserved cysteine stands out, although the primary motif associated with knottins is longer. Moreover, secondary cysteine motifs are only detectable in the enrichments associated with the two-layer sandwich of CATH and TM Structural Cluster 1. The biological meanings of the conserved cysteine need to be investigated by further biological and computational experiments.

As for the enrichment associated with anti-Gram-negative/anti-Gram-positive activities, its sequence motif is rich in positively charged amino acids. These conserved arginine and lysine residues appear periodically, supporting an amphiphilic character.

#### 2.4.2. Associated Pfam Families or Domains

Regardless of structure classification, the Pfam [12] associated with the structure–function enrichments with antifungal activities consistently include γ thionin, arthropod defensin, scorpion short toxin, and toxin-like domain. In addition, knottins are associated with cyclotides and antifungal peptide families; TM Cluster 1 and the two-layer sandwich structures contain diapausin-related AMPs. As for those associated with anti-Gram-negative/anti-Gram-positive activities, the C-terminal lipopolysaccharide-binding domain of CAP18 and the glucagon-related peptide hormone are frequently found.

## 3. Discussion

We demonstrate that there exist inclinations between structures and functions with respect to AMPs. The most universal AMP structure–function enrichments we observed happened to be antifungal activities associated concurrently with CATH’s two-layer sandwich, SCOP’s knottin, and TM’s Cluster 1. Surprisingly, CATH, SCOP, and TM could all detect this important antifungal association, suggesting the association was strong enough under any structural classification. Figure 1 further shows the high AMP sequence similarities among the three associated structures, indicating that these structures were alike in their make-up despite being obtained through three different classification approaches. The motifs with conserved cysteine and glycine, which they all had, similar to the γ-core motif of antifungal plant defensins [6], might hold the key to the antifungal activities of peptides.

Like these different motifs, the three structure–function associations were not the same. One of the most noticeable differences was SCOP’s knottins, characterized by a distinguished “disulfide through disulfide knot”, that remain significant, with antifungal activities at a low sequence identity. The peptide knots used to be thought of as rare but now have been found to be widely distributed in eukaryotic organisms [13]. We speculate that both natural and artificial knottins could be potential pharmacological agents. Although knottins are known to possess several functions such as analgesics, anthelmintic, antimalarial, and antimicrobial activities [14], this is the first study to reveal enriched antifungal activities within knottins and enriched knottins among the AMPs with antifungal activities.

As for TM Cluster 3, enriched with anti-Gram-negative/anti-Gram-positive activities, the positively-charged amphipathic AMPs, such as arginine, may appeal to negatively charged lipopolysaccharide of the outer membrane of Gram-negative bacterial cell walls as well as the negatively charged teichoic acid of Gram-positive bacterial cell walls to interact with.

As for one-direction enrichment, the inclination of TM Cluster 4 toward antiviral activities, and not the opposite, indicates that the antiviral activities of AMPs need not be the structures of TM Cluster 4, although several AMPs of TM Cluster 4 possess antiviral activities. Interestingly, the one-way inclination of antibacterial activities toward mainly alpha proteins suggests that alpha helixes alone are not destined for antimicrobial functions, and there are other major contributing factors, such as amphipathicity, to antibacterial activities.

This study has some limitations. First, the scope of structural classification in this study is restricted to CATH’s architecture and SCOP’s fold. We cannot determine any structural preferences higher than architecture/fold, such as topology/superfamily, due to the limited number of AMPs. For further structural exploration, more AMPs are required to pass statistical significance. Second, some activities of AMPs may have been misannotated or not validated experimentally. We not only examined all the AMP annotations carefully but also focused on mutual structure–function enrichments to eliminate minor or less relevant relationships.

Understanding AMP structure–function enrichments could lead to identifying novel AMPs or designing potent and therapeutic peptides as antibiotics alternatives. Our findings may boost future studies in different AMP areas, such as de novo computational design of AMPs [15,16]. For example, we may take advantage of the structural template of TM Cluster 3, as well as the tendencies of amphipathicity and net charge [17], to design novel synthetic AMPs with potent anti-Gram-negative/anti-Gram-positive activities.

## 4. Materials and Methods

### 4.1. Antimicrobial Peptides

Briefly, 3061 nonredundant experimentally-verified AMPs used in this study were obtained by combining 2774 sequences of the Collection of Anti-Microbial Peptides (CAMPR3) [18], 2619 sequences of the Antimicrobial Peptide Database 3 (APD3) [19], and 3152 sequences of A Database of Anti-Microbial Peptides (ADAM) [2]. The 3061 AMP sequences are available at http://bioinformatics.cs.ntou.edu.tw/ADAM.

Each AMP was annotated with at least one and at most seven different activities: antibacterial, anti-Gram-positive bacterial, anti-Gram-negative bacterial, antifungal, antiviral, antiparasitic, and anticancer.

### 4.2. Antimicrobial Peptide Structures

By running the compiled 3061 AMP sequences against the Protein Data Bank (PDB) [20], 445 AMP structures were identified.

The AMP structures in this study were annotated by three structural classification approaches—CATH (v4.2) [8], SCOPe (v2.07) [9], and TM. Not every AMP structure has CATH or SCOP annotations, but each AMP structure can be classified into one of the 134 TM structural fold clusters.

### 4.3. Representative Antimicrobial Peptides

The Many-Against-Many Sequence Searching Tool 2 (MMseqs2) was used to obtain AMP families by clustering highly similar sequences together and to select one representative AMP sequence for each family [21]. Using sequence identity thresholds that ranged from 100% to 40%, seven representative AMP sets were generated.

### 4.4. Structure Classification Methods

Without further specification, only the structures to which at least five original AMPs belonged were examined. All we could examine were up to the second levels of CATH or SCOP, for any higher level would fail to reach statistical significance due to the limited sample size. 

#### 4.4.1. CATH Structural Classification

There are four basic levels of CATH structural hierarchy. At the Class (C) level, proteins are divided into four categories based on secondary structure composition: mainly alpha, mainly beta, alpha–beta, and a few secondary structures. Most proteins fall into the former three categories. At the Architecture (A) level (the second CATH level), the group proteins are based on spatial arrangements of secondary structures. For example, orthogonal bundle, two-layer sandwich or alpha-beta barrel. At the Topology (T) level (the third level), the order of secondary structures is used to classify protein folds with specific structural characteristics. At the Homologous superfamily (H) level (the fourth CATH level), proteins are assigned to the same superfamily if their sequences and structures are highly similar, suggesting a plausible common evolutionary ancestor [8,22].

CATH explores protein structure–function relationships according to protein evolution. Functional Families (Fun-Fam), which tend to have highly similar structures and functions, are formed by clustering protein domains (sequences and structures) within homologous superfamilies [8].

#### 4.4.2. SCOP Structural Classification

The five basic levels of the SCOP database are Class, Fold, Superfamily, Family, and Domain. The SCOP database used to rely heavily on manual classification, with a focus on protein evolution and structure similarities.

Instead, the SCOPe (SCOP-extended) database [9], which inherited the SCOP database and corrected some classification errors by integrating the Astral database, has become more automatic. Like the first CATH level, SCOP Class only considers the overall secondary structure composition. There are 12 classes in the SCOPe Class, while most proteins are assigned to five classes, namely, all alpha, all beta, alpha and beta proteins (α/β) that contain α-helices and β-folds, alpha and beta proteins (α + β) with separate α-helices and β-folds, and small proteins. SCOP Fold further divides proteins by topology and architecture. It is not until the second level that the divergence between CATH and SCOP becomes apparent. The SCOP Superfamilies are more distantly related than the Families, while each Superfamily contains all the domains in a fold, and the proteins within the same SCOP Family, which share at least 30% sequence identity, often have distinct functions. There are two additional levels above Domain in the SCOPe: Species and Protein Domain. Species represents a distinct protein sequence derived from a specific source, while Protein Domain gathers isoforms or similar sequences from any source.

#### 4.4.3. TM Structural Classification

To overcome the drawback of conventional protein structural classification methods, the TM structural classification introduces TM-scores [10]. Conventional structural classification often fails to recognize similar protein structures with different lengths, for it relies on calculating the length-sensitive root mean square deviation (RMSD). To evaluate protein structure similarity regardless of their lengths or sizes, the TM method adopts a geometric goodness of fit approach (the TM-score), which utilizes the Levitt–Gerstein weight factor that prefers close residue pairs to distant ones.

The TM-score of two structures ranges from 0 to 1. When the TM-score ≥0.5, the proteins are considered to fall into identical topological structures; otherwise, the proteins have different structures [23].

The order of the TM clusters used in this study is adopted from ADAM [2].

### 4.5. Enrichment Analysis

The enrichment analysis uses the hypergeometric test to check whether a specific property is over-/under-represented in a sample based on the hypergeometric distribution. If the number of objects with the specific property is significantly greater than expected compared to the population, it is enriched or over-represented; otherwise, it is not. To check whether a specific property is enriched in a sample, the following formula is used:

(1) $$P=\overset{n}{\underset{i=m}{\sum }}\;\;\frac{\left(\begin{array}{c} M\\ i \end{array}\right)\;\left(\begin{array}{c} N-M\\ n-i \end{array}\right)}{\left(\begin{array}{c} N\\ n \end{array}\right)}$$

where *N* is the population size, *n* is the sample size, and *M* and *m* are the numbers of objects with the specific property in the population and in the sample, respectively. The *p*-value, which refers to the probability of at least m objects occurring in the sample, must be <0.05 for enrichment. Due to multiple hypotheses, the *p*-values are adjusted by Benjamini–Hochberg correction [11].

We examined two different concepts using enrichment analysis. First, we examined whether there are any antimicrobial functional tendencies within a specific structure, where in terms of the number of AMP sequences, *N* and *M* are the population size with any functions and with a specific function, respectively, while *n* and *m* are the sample size of the specific category of structures with any functions and with the specific function, respectively. Second, we examined whether there are any structural tendencies for a specific antimicrobial function, where *N* is the population size under a structural classification, *M* is the subpopulation size with a specific category of structures under this classification, and *n* and *m* are the sample size of a specific antimicrobial function with any structures and with the specific structure, respectively. The population size and sample size of the enrichment analyses are detailed at Supplementary Files S1 and S2.

## 5. Conclusions

Structural tendencies and functional tendencies exist with respect to AMPs. The structure–function mutual tendency happens to antifungal activities of AMPs with the two-layer sandwich architecture of CATH, the first structural cluster of TM, and the knottin fold of SCOP. This study gives hints on how to apply protein engineering to design AMPs as therapeutic agents by modifying their sequences and structures.

## Figures and Tables

**Table 1:** Significant enrichments between functions and structures of antimicrobial peptides.

Type	Structure		Sequence Identity Threshold (%)							F(x)
	1st Level	2nd Level	100	90	80	70	60	50	40	
**CATH**	α β	*	⟷							F
		2-Layer Sandwich	⟷	⟷	⟷			→		F
		Roll	←							G
		Roll	←							G-
	Mainly α	*	←	←	←	←				G
		*	←							G+
		Orthogonal Bundle	←							G
	Mainly β	*	→							P
		Beta Barrel	←							G-
**SCOP**	α and β (α + β)	*	←							G
		*	←							G+
		IL8-like	⟷	←	←			←		G-
	Peptides	*	→							G
		*	→							G+
	Small proteins	*	⟷	⟷	⟷	⟷	⟷	⟷	⟷	F
		Knottins	⟷	⟷	⟷	⟷	→	⟷	→	F
		Crambin-like	⟷	⟷	⟷					C
		Defensin-like	←							G-
**TM**	TM1		⟷	⟷	⟷	→	⟷	⟷	→	F
	TM2		⟷	⟷	⟷	⟷	→	⟷	⟷	G-
	TM3		⟷							G
	TM3		⟷	⟷	⟷	⟷	←	⟷	→	G+
	TM3		⟷	⟷	⟷	⟷	⟷	⟷	⟷	G-
	TM4		←							G
	TM4		←							G+
	TM4		←							G-
	TM4		⟷	→	→	→			→	V
	TM5		⟷	⟷	→	→				V

Abbreviations: C, anticancer; F, antifungal; G, antibacterial; G+, anti-Gram+; G-, anti-Gram-; P, antiparasitic; V, antiviral; F(x), function; →, enriched function; ←, enriched structure; ⟷, structure–function mutual enrichment; *, all the structures within the classification. The Benjamini–Hochberg correction [11] was applied to adjust *p*-values at Q < 0.05.

## Supplementary Materials

The following are available online at https://www.mdpi.com/1422-0067/21/22/8783/s1. File S1: The population size *N* and *M* in the enrichment analyses. File S2: The sample size *n* and *m* in the enrichment analyses.

## Abbreviations

ADAM	A Database of Anti-Microbial Peptides
AMP	Antimicrobial peptide
CATH	Class, Architecture, Topology, Homology
SCOP	Structural Classification of Proteins
TM	Template Modeling
